# Navigation path extraction for inter-row robots in Panax notoginseng shade house based on Im-YOLOv5s

**DOI:** 10.3389/fpls.2023.1246717

**Published:** 2023-10-17

**Authors:** Yu Tan, Wei Su, Lijun Zhao, Qinghui Lai, Chenglin Wang, Jin Jiang, Yongjie Wang, Peihang Li

**Affiliations:** ^1^Faculty of Modern Agricultural Engineering, Kunming University of Technology, Kunming, China; ^2^College of Intelligent and Manufacturing Engineering, Chongqing University of Arts and Sciences, Chongqing, China; ^3^School of Energy and Environmental Science, Yunnan Normal University, Kunming, China

**Keywords:** computer vision, Improved YOLOv5s, agricultural robot, navigation line extraction, seven-fork root detection

## Abstract

**Introduction:**

The accurate extraction of navigation paths is crucial for the automated navigation of agricultural robots. Navigation line extraction in complex environments such as Panax notoginseng shade house can be challenging due to factors including similar colors between the fork rows and soil, and the shadows cast by shade nets.

**Methods:**

In this paper, we propose a new method for navigation line extraction based on deep learning and least squares (DL-LS) algorithms. We improve the YOLOv5s algorithm by introducing MobileNetv3 and ECANet. The trained model detects the seven-fork roots in the effective area between rows and uses the root point substitution method to determine the coordinates of the localization base points of the seven-fork root points. The seven-fork column lines on both sides of the plant monopoly are fitted using the least squares method.

**Results:**

The experimental results indicate that Im-YOLOv5s achieves higher detection performance than other detection models. Through these improvements, Im-YOLOv5s achieves a mAP (mean Average Precision) of 94.9%. Compared to YOLOv5s, Im-YOLOv5s improves the average accuracy and frame rate by 1.9% and 27.7%, respectively, and the weight size is reduced by 47.9%. The results also reveal the ability of DL-LS to accurately extract seven-fork row lines, with a maximum deviation of the navigation baseline row direction of 1.64°, meeting the requirements of robot navigation line extraction.

**Discussion:**

The results shows that compared to existing models, this model is more effective in detecting the seven-fork roots in images, and the computational complexity of the model is smaller. Our proposed method provides a basis for the intelligent mechanization of Panax notoginseng planting.

## 1 Introduction

Panax notoginseng is a valuable Chinese herbal medicine with numerous medicinal properties, and its cultivation has increased in recent years. However, the production process still relies on outdated technology, and there is a need for efficient and intelligent production methods to improve productivity. One potential solution is the use of mechanized and intelligent agricultural equipment such as robots, which can replace manual labor and increase the scale of cultivation (Cheng et al., 2023). In the semi-structured planting environment of Panax notoginseng shade house, the real-time and accurate extraction of robot navigation paths is essential for autonomous robot navigation.

Automated navigation techniques used for unstructured environments, such as large fields and orchards, mainly include satellite positioning navigation, Light Detection and Ranging (LiDAR) navigation, and visual navigation (Zhang et al., 2020; Zhou and He, 2021). However, the strong shading effect of shade nets in the Panax notoginseng shade house environment renders commonly used navigation systems including Global Positioning System (GPS) and BeiDou Navigation Satellite System (BDS) ineffective due to poor signal quality (Gai et al., 2021). LiDAR-based navigation requires high computational power, which makes the extraction of navigation features difficult and results in high equipment costs (Bai et al., 2023). On the other hand, visual navigation acquires imagery through cameras and uses techniques such as image processing, deep learning, and navigation feature target detection to obtain navigation lines. This method is able to provide multiple levels of detection information, is low-cost, and has a high real-time performance and wide applicability (Wang T. et al., 2022). In highly occluded environments, vision-based navigation is the mainstream method used to obtain interline navigation information (Radcliffe et al., 2018). In particular, vision-based robot navigation techniques are widely used in research on fields, orchards, and forests. For such applications, the precise positioning of the crops in the image is the basis for the accurate extraction of navigation lines. The most commonly used methods adopted for the extraction of navigation lines in agricultural machinery typically include several processing steps for navigation line extraction, such as the 2G-R-B grayscale, Otsu binarization of images, vertical projection, and Hough transform (Chen et al., 2020; Chen et al., 2021). These methods are based on the large difference between the crop color and the background color, which facilitates the use of image processing methods to extract navigation lines (Zhang et al., 2017). Research on visual navigation for orchards and forests generally focuses on road or sky-based navigation line generation (Opiyo et al., 2021) and crop detection-based navigation line fitting (Su et al., 2022), depending on the type, shape, and height of the plants. Crop detection-based navigation methods require the accurate identification of crop trunks and are highly robust to complex road environments, and therefore demand high adaptability (Juman et al., 2016). Furthermore, although the above algorithm can identify the center line of crop rows, the identification conditions are relatively simple and are not able to account for different growing environments and external disturbances.

The ability of traditional image processing methods to distinguish between scenes with similar backgrounds and targets is reduced due to their susceptible to light, canopy cover, and weeds. However, deep learning methods can extract features beyond our understanding for object detection. In recent years, the development of artificial intelligence and computer hardware has facilitated the deployment of deep learning models on embedded devices (Aguiar et al., 2020). Moreover, computer vision-based detection methods are less costly compared to traditional detection approaches. As a result, deep learning-based methods have gained widespread attention for the extraction of navigation features. For example, Ma et al. (2021) used Faster R-CNN to construct a target detection model for the trunk recognition of the effective distance between the rows of an orchard. The model was able to extract navigation lines based on cubic spline interpolation and subsequently realized the generation of navigation lines between the rows of a kiwifruit orchard, providing a new reference for orchard navigation. Li et al. (2022) employed LiDAR point cloud data to identify obstacles such as rocks and soil blocks between rows, obtaining auxiliary navigation data to supplement the visual information and improve the recognition accuracy of inter-row navigation data in mid- and late-season maize. Zhou et al. (2022) used YOLOv3 to identify orchard trunks and fruit trees and adopted the least squares method to fit a reference line with growth on both sides, achieving a 90% accuracy in extracting the orchard center line. However, the authors did not integrate the detection results with path planning, and deployment on embedded hardware was not considered. Shanshan et al. (2023) combined an improved YOLOv5 network with an improved centerline extraction algorithm to detect straight and curved crop rows, yet the method is only applicable to the seedling stage of rice. The aforementioned deep learning-based navigation methods can solve the real-time and robustness problems of navigation in numerous scenarios, however they are not effective for the navigation problem in the Panax ginseng shade house environment. Thus, in order to fulfill the needs of embedded device applications and enhance navigation accuracy, the model size, accuracy, and frame rate of the model proposed by Wang et al. requires improvement. Comprehensive and in-depth research on navigation line extraction for Panax notoginseng shade house is limited. However, it is possible to adopt the navigation methods used in orchard and forest visual detection to obtain navigation feature points based on deep learning. To achieve this, it is necessary to ensure that the model is small enough, has anti-interference capabilities, and is highly accurate for deployment on embedded devices and to meet the operational requirements of robots.

**Figure 1:**
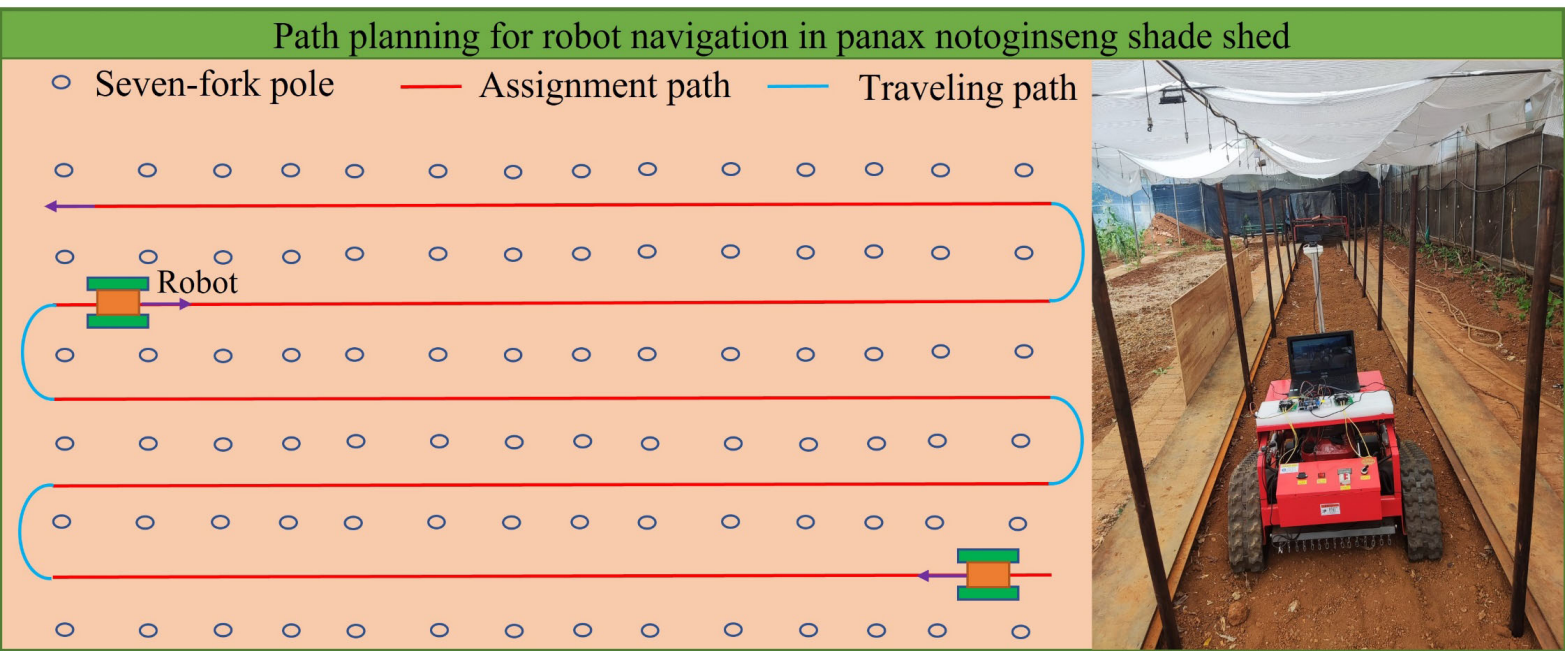
Navigation path planning map.

Therefore, in this paper, based on the environment inside Panax pseudoginseng shade house, we address the bottlenecks associated with robot navigation line extraction algorithms between the rows of the shade house in the complex farmland environment, including poor effects and adaptability. In particular, we propose a method that combines deep learning and least squares (DL-LS) algorithms to obtain the inter-row navigation lines in Panax pseudoginseng shade house. In order to improve the detection accuracy and speed, we take the position of the root point of seven branches as the main navigation information, and propose a lightweight network model with improved YOLOv5s architecture to identify the roots to accurately identify navigation lines in the complex shade house environment. **Figure 1** describes the navigation path planning of the robot working in the Panax notoginseng shade house, with a focus placed on extracting the middle red navigation line. The proposed method provides a new and effective navigation approach for Panax notoginseng shade house, which can act as a guide for the intelligent mechanized operation of this species. The main contributions are summarized as follows:

(1) Based on YOLOv5’s target detection model, we weaken the backbone network by replacing the original backbone with MobileNetv3, and introduce the ECANetention mechanism module to pay more attention to the seven-branch root characteristics.
(2) Verifying the effectiveness of the improved YOLOv5s by an ablation study and comparing it with other mainstream single-stage target detection models.
(3) We use the improved YOLOv5s model to locate the small area of the seven-fork root within the region of interest (ROI) in the video and extract the coordinates of the midpoint of the lower bottom frame line rather than using the root point. We then combine the least squares method to fit the tree line on both sides and use the angle tangent formula to extract the traverse navigation line for the robot.
(4) Establishing a new dataset of shade house environments, and the proposed method was tested and analyzed using a built data acquisition robot.

The remaining part of this paper is organized as follows. The second section discusses the navigation line extraction method, the improved YOLOv5s, and the evaluation metrics. The third section presents the robot platform and the experimental results, the fourth section shows the discussion, and the fifth section summarizes the conclusions.

## 2 Materials and methods

### 2.1 Navigation line generation process

**Figure 2:**
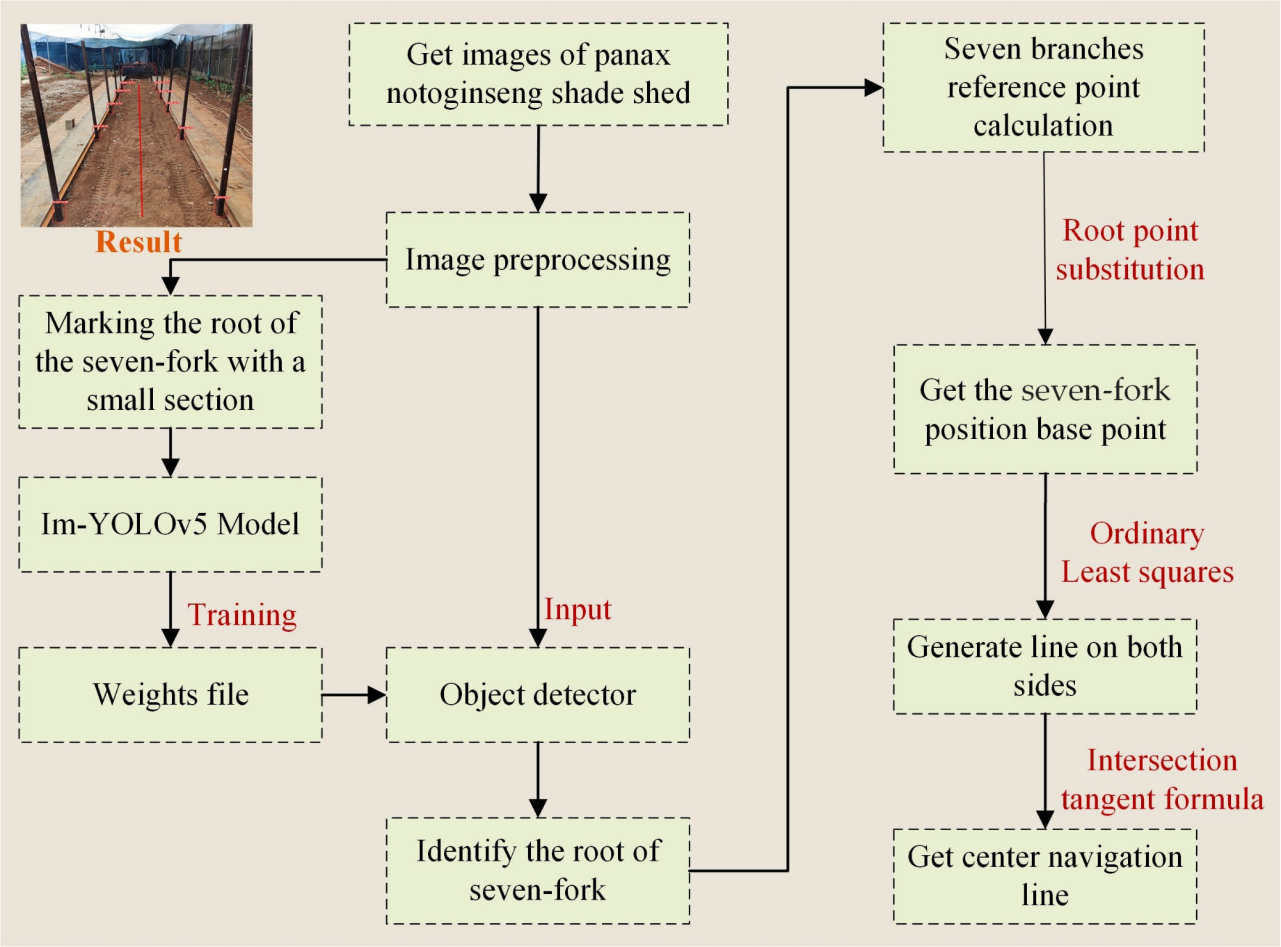
Flowchart of the proposed extraction method for Panax shade house navigation line.

**Figure 2** depicts the extraction process of inter-row navigation lines within the Panax notoginseng shade house, which includes the following key steps: 1) The acquired images are preprocessed by cropping redundant parts and performing data expansion. 2) The Im-YOLOv5s network is trained using manually labeled seven-fork root feature maps. The weight files are generated and the seven-fork root detection model for the Panax notoginseng shade house is obtained. 3) The trained detection model is used for the inter-row seven-fork root detection. By determining the center coordinates of the bottom frame using the key coordinate information of the rectangular frame, we generate the localized base point coordinates of the detected trunk based on the root point substitution method. 4) The least squares method is used to fit the inter-monopoly seven-fork column lines on both sides based on the positioning base point coordinates. 5) The navigation lines are extracted based on the navigation base lines on both sides using the angle tangent formula.

### 2.2 Image acquisition and pre-processing

**Figure 3:**
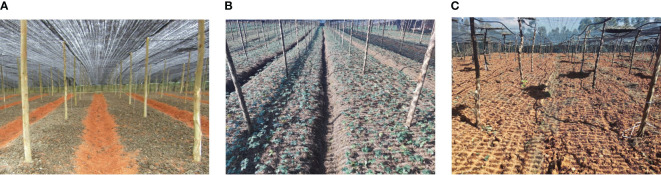
Pretreated results among different rows scenes. **(A)** Land to be sown; **(B)** seedling land; **(C)** harvested land.

Traditional Panax notoginseng shade house are typically constructed using seven-fork structures with diameters ranging from φ5 to φ8 cm. They are generally planted based on a grid of 2.4 m × 2.0 m (length × width) dimensions, with a 1.8 m scaffold height and a shade net covering the top layer to provide uniform light transmission. The test pictures were taken on November 9, 2022, in a Panax notoginseng shade net plantation in Shilin Yi Autonomous County, Kunming City, Yunnan Province, China. The plantation included a seedling plot, a plot to be sown, and a shade house planting site just after harvest. For the image acquisition, a COMS camera was mounted horizontally on a robotic platform 1.4 m above the ground and placed in the row center. We collected a total of 412 images from three scenes under different angles and lighting conditions: the Panax notoginseng sowing field; the Panax notoginseng harvesting field; and the Panax notoginseng seedling field. **Figure 3** presents images of the three different scenes. To minimize the interference of trunks in the non-row inspection area and to improve the training speed and accuracy of the detection model, we preprocessed the images by cropping the non-row redundant parts. After several cropping comparisons, we determined that uniformly converting the input image resolution to 2,000×1,000 allows us to identify interlinear information in the sample images of the different scenes.

### 2.3 Training sample labeling

To improve the robustness of the model and suppress overfitting, we added random perturbations such as saturation, flipping, and luminance during the training process. This expanded the amount of available information and enhanced the richness of the experimental data. As a result, the 412 images were expanded to 936 images and divided into training and validation sets with a ratio of 8:2. Moreover, we used 180 images captured by an external computer camera as the test set to evaluate the performance of the model during the training process. The test set was not involved in the actual training. In each example image, the roots of the seven-forks were marked by rectangular boxes and LabelImg installed on Anaconda was used for the image labeling. To ensure labeling efficiency and accuracy, we only labeled two rows of hepta-roots within 12 m of the capture point. Each side of the tree rows contained 3–5 labeled hepta-roots. A total of 936 images were labeled, resulting in 7,288 labeled hepta-roots, which were saved as label files in XML format. This labeling process was based on a robot walking speed of 0.5–1 m/s.

### 2.4 Improved YOLOv5s network

The YOLOv5s model is a lightweight version of You Ony Look Once (YOLO) algorithm with fewer layers, allowing for a faster detection. Therefore, the aim of this paper is to apply the improved model to the detection of seven-fork roots based on the YOLOv5s model. The Im-YOLOv5s is improved by reducing its backbone network using MobileNetv3 and introducing the ECANet attention mechanism module to enhance the extraction of useful information and compress useless information. This enhances the recognition accuracy and robustness of the model. With these improvements, the model can efficiently, accurately, and quickly obtain the root information of the seven-fork in the shadow trellis while reducing the weight size, which is convenient for use in embedded devices.

#### 2.4.1 YOLOv5s network

**Figure 4:**
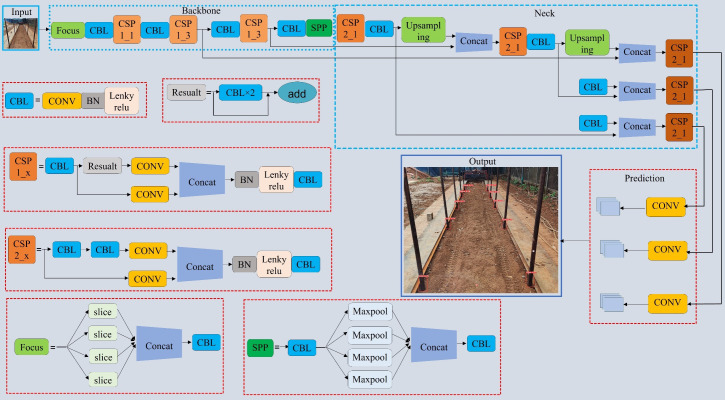
YOLOv5s network structure.

The YOLOv5 target detection model is known for its faster detection speed and smaller model size with guaranteed accuracy, making it an ideal choice for efficiently detecting the seven-fork roots in this study. The YOLOv5 model is divided into four variants: YOLOv5s; YOLOv5l; YOLOv5m; and YOLOv5x (Zhang et al., 2022). YOLOv5s is the smallest in terms of depth and feature map width. In order to ensure accuracy while contributing to real-time detection and reducing the model size, we made some improvements to the YOLOv5s target detection network. The network structure of YOLOv5 consists of four parts, namely, Input, Backbone, Neck, and Prediction. The size of the model directly affects its deployment on mobile devices and real-time detection. Compared to other algorithms, YOLOv5 has advantages in terms of speed and model size. Considering the characteristics of the dataset, the number of parameters, and the training time, we chose the lightest model, YOLOv5s. **Figure 4** presents the network structure of YOLOv5s. The backbone is composed of two key components, C3 and CONV, and contains a large number of convolutional layers, which were mainly improved in this study via the components in the blue dashed box in **Figure 4**.

#### 2.4.2 Improvement based on MobileNetv3

**Figure 5:**
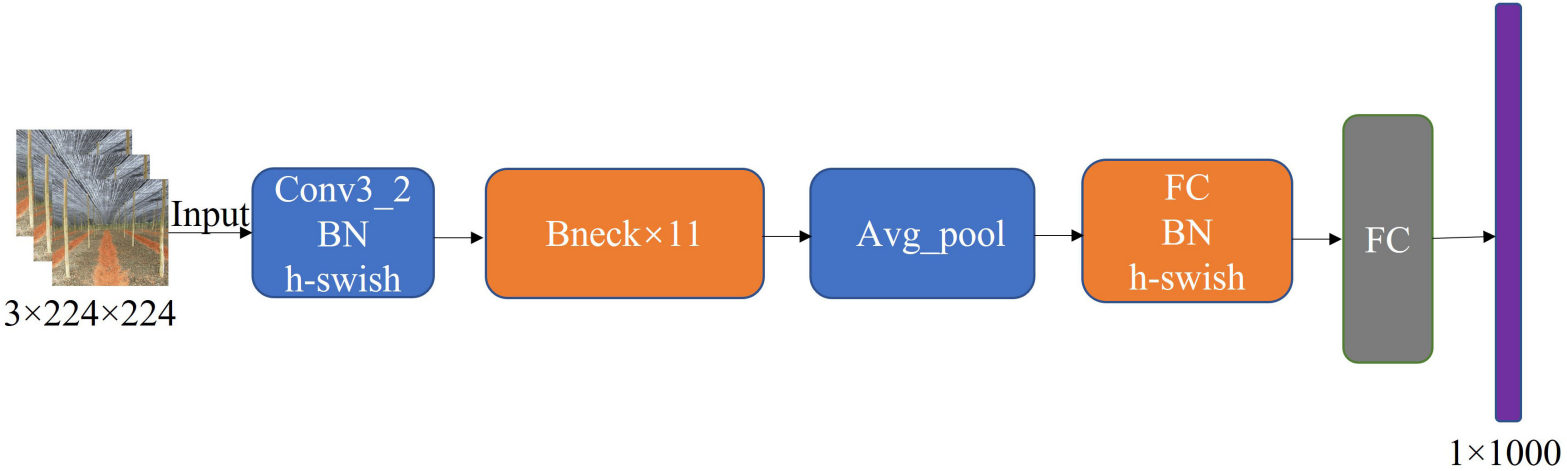
MobileNetv3 network architecture.

A large number of convolutional layers increases a model’s memory footprint. This is not conducive to deploying the model on embedded devices. Compared to heavyweight networks, lightweight networks have fewer parameters, require less computation, and have a shorter inference time. Lightweight networks are thus more suitable for scenarios with limited storage space and power consumption, such as embedded terminals, robots, and other small systems. MobileNetv3 (Howard et al., 2019) is the third generation of lightweight networks released by Google in 2019, designed for devices with limited memory and computation. MobileNetV3 is a successor of MobileNetV1 (Howard et al., 2017) with deep separable convolution and MobileNetV2 (Sandler et al., 2018). It adds neural network architecture search (NAS) and h-swish activation functions, and introduces the squeeze-and-excitation channel attention mechanism (SE) to improve both performance and speed. MobileNetV3 has two versions, Large and Small, for high and low resource scenarios, respectively. The overall structure of the versions is the same, with the difference being in the number of basic units bottleneck and internal parameters. **Figure 5** presents the network structure of MobileNetv3. In this paper, we used the MobileNetv3-Small lightweight network instead of the YOLOv5s backbone network to extract the seven-fork root images with effective features based on the actual scenario. We compared the Im-YOLOv5 network with the introduction of MobileNetv3-Small to the original YOLOv5s network, revealing a 28% reduction in parameters from 7,022,326 to 5,024,100.

#### 2.4.3 Introducing the attention mechanism

**Figure 6:**
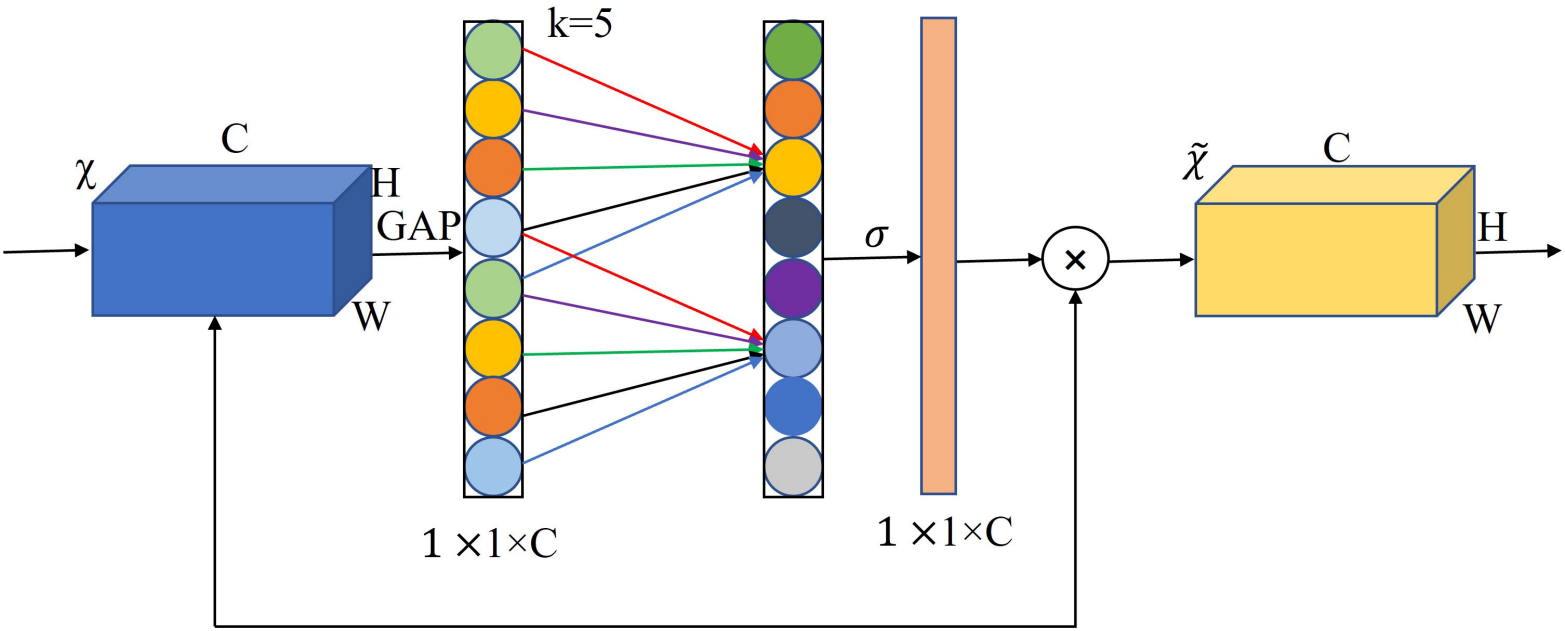
ECANet channel attention.

The channel attention mechanism has the potential to greatly improve the performance of deep convolutional neural networks (CNNs). However, while SE downscaling can reduce model complexity, it destroys the direct correspondence between channels and their weights. To overcome the trade-off between performance and complexity, and to improve the accuracy and efficiency of the algorithm for seven-fork root detection in a three-seven shade house environment, we introduce an efficient channel attention (ECA) module (Xue et al., 2022) into the lower neck structure of MobileNetV3-Small. This module enables the network to pay different levels of attention to different channel features, giving more weight to important feature channels and less weight to irrelevant feature channels. This allows the algorithm to compress useless information and improve detection accuracy. **Figure 6** depicts the ECANet structure.

**Figure 7:**
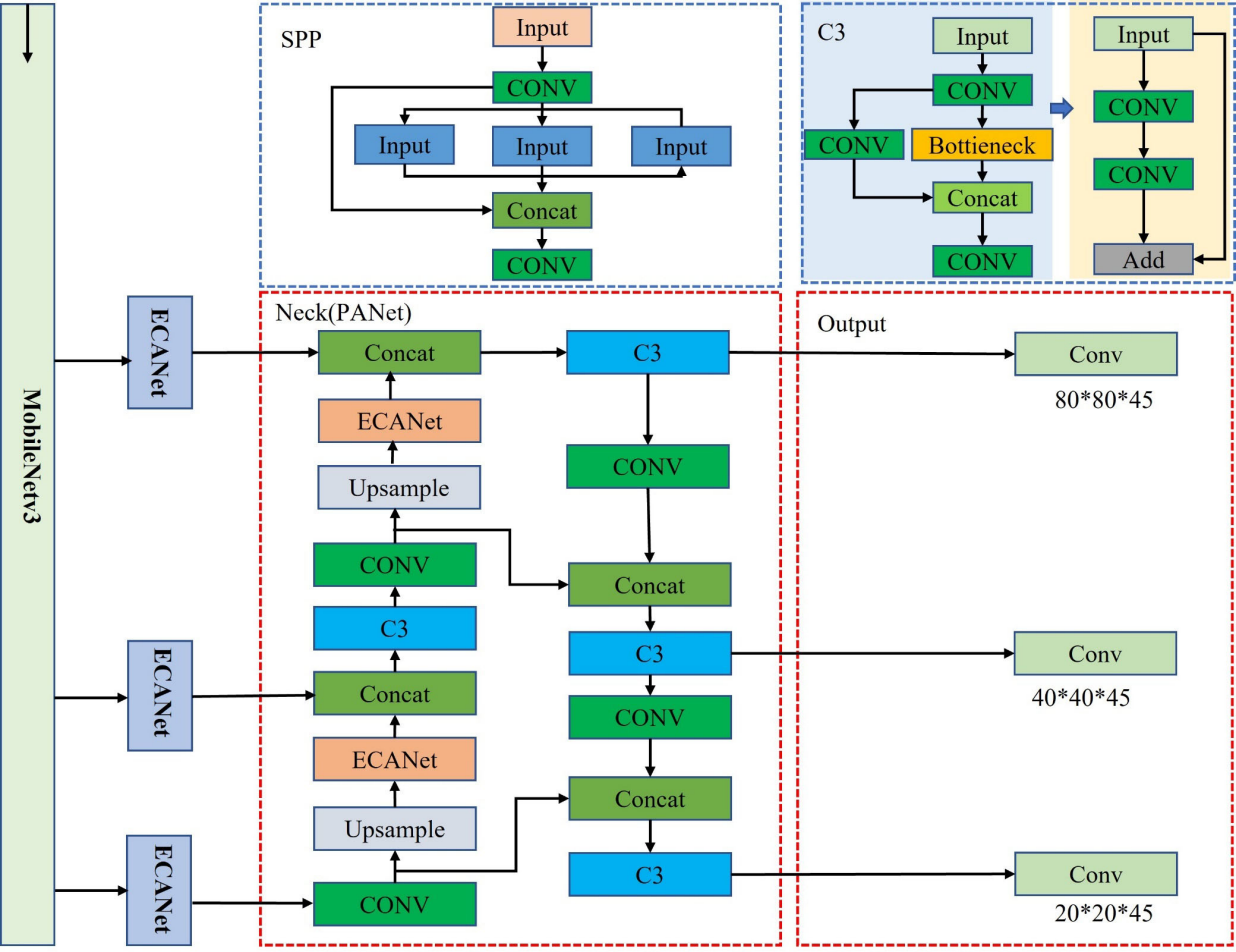
Improved YOLOv5s architecture.

In this study, we used MobileNetV3 as the backbone model and combined YOLOv5s with the ECANet and CBAM modules to perform seven-fork root detection experiments. **Table 1** reports the experimental results. ECANet outperformed CBAM, indicating that ECANet can improve the performance of YOLOv5s at a lower cost. In addition, ECANet is more competitive than CBAM as it offers a higher accuracy and lower model complexity. **Figure 7** presents the specific structure of the Im-YOLOv5 algorithm.

**Table 1:** Comparison of the recognition performance of YOLOv5 with different modules.

Method	P(%)	R(%)	Model size (MB)	FPS	mAP(%)
YOLOv5s	91.0	91.4	14.4	83.3	93.1
MobileNetv3	92.0	90.8	10.5	73.5	92.9
MobileNetv3+ECANet	94.2	92.0	7.5	83.3	94.9
MobileNetv3+CBAM	93.9	91.0	10.5	64.1	93.6

#### 2.4.4 CIoU loss algorithm

The Complete Intersection over Union (CIoU) accounts for the overlapping area, height, and centroid distance of the target and prediction boxes, which addresses the shortcomings of the Generalized Intersection over Union (GIoU) loss function. This results in a more stable regression equation for the target box, with a faster convergence speed and higher convergence accuracy. Therefore, we used the CIOU_Loss function rather than the GIOU_Loss function for the bounding box loss in Im-YOLOv5. To calculate the loss of class probability and the target score, we employed the binary cross-entropy and logit loss functions (Gui et al., 2023), respectively, defined as follows:


(1) $$GIoU=I\text{o}U-\frac{\left|C-\left(\text{A}\cup B\right)\right|}{\left|C\right|}$$

(2) $$I\text{o}U=\frac{\left|\text{A}\cap B\right|}{\left|\text{A}\cup B\right|}$$

(3) $$CIoU=1-IoU+\frac{\rho^{2}(\text{A},B)}{c^{2}}+\alpha *\nu$$

(4) $$\alpha =\frac{v}{(1-IoU)+V}$$

(5) $$v=\frac{4}{\pi^{2}}{\left(\arctan\frac{\omega^{gt}}{h^{gt}}-\arctan\frac{\omega }{h}\right)}^{2}$$

where A is the prediction box; B is the ground truth box; C is the smallest box that completely encloses A and B; ρ(A,B) is the Euclidean distance between the center coordinates of boxes A and B; c is the diagonal distance of the smallest box that encloses boxes A and B; α is the weight function; ν is the function that measures the consistency of the aspect ratio; $w^{gt}$ and $h^{gt}$ are the width and height of the ground truth box, respectively; and w and h are the width and height of the prediction box, respectively.

#### 2.4.5 Model evaluation

In this paper, we employed five metrics, namely precision (P), recall (R), mean average precision (mAP), model size, and detection speed, to evaluate the seven-fork root detection model. A true positive case indicates that the intersection over union (IoU) is greater than or equal to 0.5; a false positive case indicates that the IoU is less than 0.5; and a false negative case indicates that the IoU is equal to 0 (Li et al., 2021). P, R, F, AP, and mAP are calculated using the equations in equations (6)–(10) in the following:


(6) $$p=\frac{TP}{TP+FP}*100\%$$

(7) $$R=\frac{TP}{TP+FN}*100\%$$

(8) $$F=\frac{2*P*R}{P+R}$$

(9) $$AP=\int_{0}^{1}P_{\left(R\right)}{\text{d}}_{R}$$

(10) $$\text{m}AP=\frac{1}{M}\sum_{\text{k}=1}^{M}\text{A}*P(\text{k})$$

where TP, FP, and FN are the number of true positives, false positives, and false negatives respectively; and M is the number of detection categories.

Furthermore, we employed frames per second (FPS) to evaluate the detection speed of different models. A higher value of FPS indicates a better real-time performance of the model. We also used giga floating point operations per Second (GFLOPs) as an evaluation indicator to measure the computational power of the model, with higher values denoting a higher demand on the machine’s computing power.

### 2.5 Model training

The models were trained on a desktop workstation with the following specifications: 64 GB of memory; an Intel Xeon^®^ W-214 CPU; and an NVIDIA RTX 2080Ti GPU with 11 GB of video memory. The workstation operated on Windows 11 (64-bit), and the training was conducted using Python 3.9 with the deep learning platform CUDA 11.6 and the Pytorch framework.

The quality of the training model is significantly influenced by the difference in training parameters, and hyperparameters such as the learning rate, batch size, and number of iterations must be set manually during the training process. Among them, the learning rate is crucial in deep learning optimizers as it determines the speed at which weights are updated. If the learning rate is too high, the training results will exceed the optimal value, while if it is too low, the model will converge too slowly. The batch size depends on the size of the computer memory, with larger batches providing better model training results. The number of iterations determines the number of training rounds, with more iterations taking longer to complete. The iteration typically ends when the loss value has fully converged. After several parameter adjustments, the parameters in the model were set according to the values provided in **Table 2**.

**Table 2:** Target detection hyperparameter settings.

Parameter	Im-YOLOv5	YOLOv5	YOLOv3	YOLOv7
Backbone network	MobileNetv3	Backbone	Darknet53	Backbone
Training size	640 × 640	640 × 640	416 × 416	640 × 640
Batch size	8	8	8	8
No.of categories	1	1	1	1
Initial learning rate	0.001	0.001	0.001	0.001
No.of iterations	300	300	300	300

### 2.6 Root point substitution method

The selection and extraction of navigation features are crucial for inter-row navigation in shade house. In this paper, we defined the root point as the midpoint of the dividing line between the seven-fork point and the ground. The root point of the fork is used as the base point for row positioning in the construction of Panax notoginseng shade house. Therefore, the root point of the fork is considered the optimal inter-row navigation feature. However, since the target color of the Panax notoginseng shade house environment is similar to the background color, it is both challenging and time-consuming to filter out other interferences using image processing methods. To address this issue, rather than using navigation base points, we proposed a heptagram-based generation method. We trained a deep learning-based seven-fork root model and used the trained detection model to generate the minimum rectangular detection frame outside the bottom of the seven-fork. The midpoint of the bottom edge of the detection frame was observed to correspond well with its root point.

### 2.7 Navigation line extraction method

Once the root points of the bottom of the seven-fork were obtained, we fitted the crop rows using the seven-fork root points of the Panax notoginseng shade house. We employed the least squares method to fit the coordinates of these root points using equations (11)–(13):


(11) $$\left\{ \begin{array}{l} \bar{X}=\frac{\sum_{\text{i}=1}^{\text{n}}x_{\text{i}}}{\text{n}}\\ \bar{Y}=\frac{\sum_{\text{i}=1}^{\text{n}}y_{\text{i}}}{\text{n}} \end{array}\right.$$

(12) $$\text{m}=\frac{\sum_{i=1}^{\text{n}}\left(x_{i}-X)*(y_{i}-Y\right)}{\sum_{i=1}^{n}{\left(x_{i}-X\right)}^{2}}$$

(13) $$b=\bar{Y}-m*\bar{X}$$

where $\bar{X}$ denotes the average of the horizontal coordinate of all root points; $\bar{Y}$ is the average vertical coordinate; $x_{i}$ and $y_{\text{i}}$ are the horizontal and vertical coordinates of each root point; respectively; i is the serial number; m is the slope; and b is the intercept. Thus, the fitted line can be expressed as y=m∗x+b .

**Figure 8:**
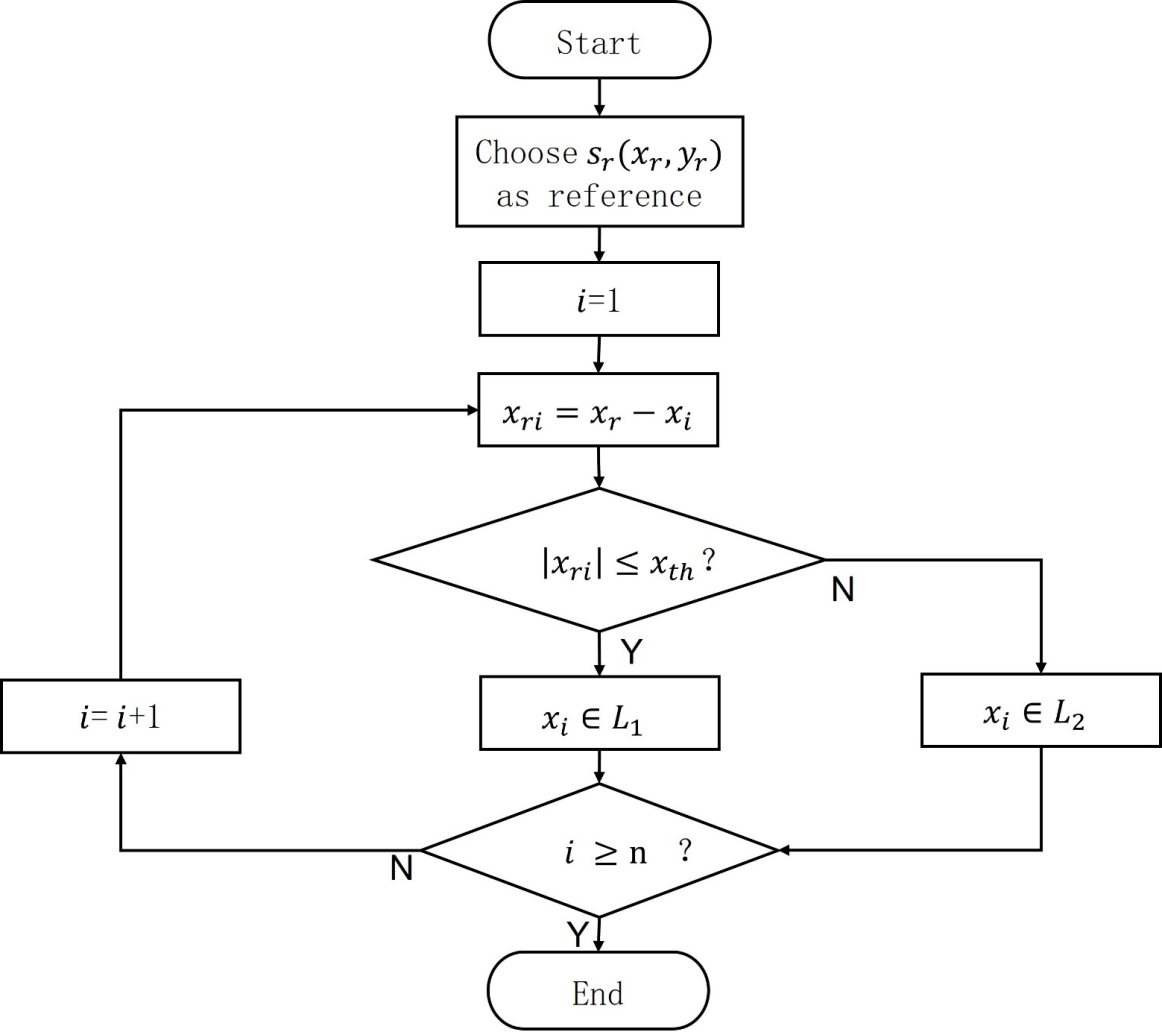
Algorithm flowchart of classification of points into different lines.

In order to obtain two lines from the detected coordinates of the root points, we separated the points using a positive threshold and a reference point. We set a positive threshold $x_{th}$ and reference point $s_{r}(x_{r},y_{r})$ i≤n , representing the sequence number, where n is the total number of points. Since the seven rows of pitchforks extend in the positive y-direction, we only used the x-values for our calculations. The two groups of points are denoted as L_1_ and L_2_. If for example, the absolute value of $\left(x_{r1}=x_{r}-x_{1}\right)$ is less than or equal to $x_{th}$ , the points $s_{1}$ are divided into L_1_ and vice versa for L_2_. **Figure 8** presents the algorithm flow. After classifying all the detected points, we calculated the values of m and b, and fitted the expression parameters for the seven-forked rows on both sides as $y_{L1}=m_{1}*x+b_{1}$ and $y_{L2}=m_{2}*x+b_{2}$ .

Once the expressions of the line parameters on both sides were determined, we used the angular bisector of the left and right seven-forked row lines as the robot navigation baseline. The principle of tangency between two lines was then adopted to obtain the robot navigation parameters via equation (14). More specifically, we calculated the robot navigation line slope m based on the relationship that the tangent angle between *m* and *m_1_* is equal to the tangent angle between m and *m_2_*:


(14) $$\frac{m-m_{1}}{1+m*m_{1}}=\frac{m_{2}-m}{1+m*m_{2}}$$

where *m* is the slope of the robot’s navigation center line; *m_1_* is the slope of the left seven-branch line; and *m_2_* is the slope of the right seven-branch line.

## 3 Experiments and results

The focus of this study is the acquisition of navigation information in the Panax notoginseng shade. The obtained navigation information can be used in later path planning stages of the robot to facilitate autonomous driving. It can also be used as a basis for adjusting the driving state of the robot.

### 3.1 Experimental platform

**Figure 9:**
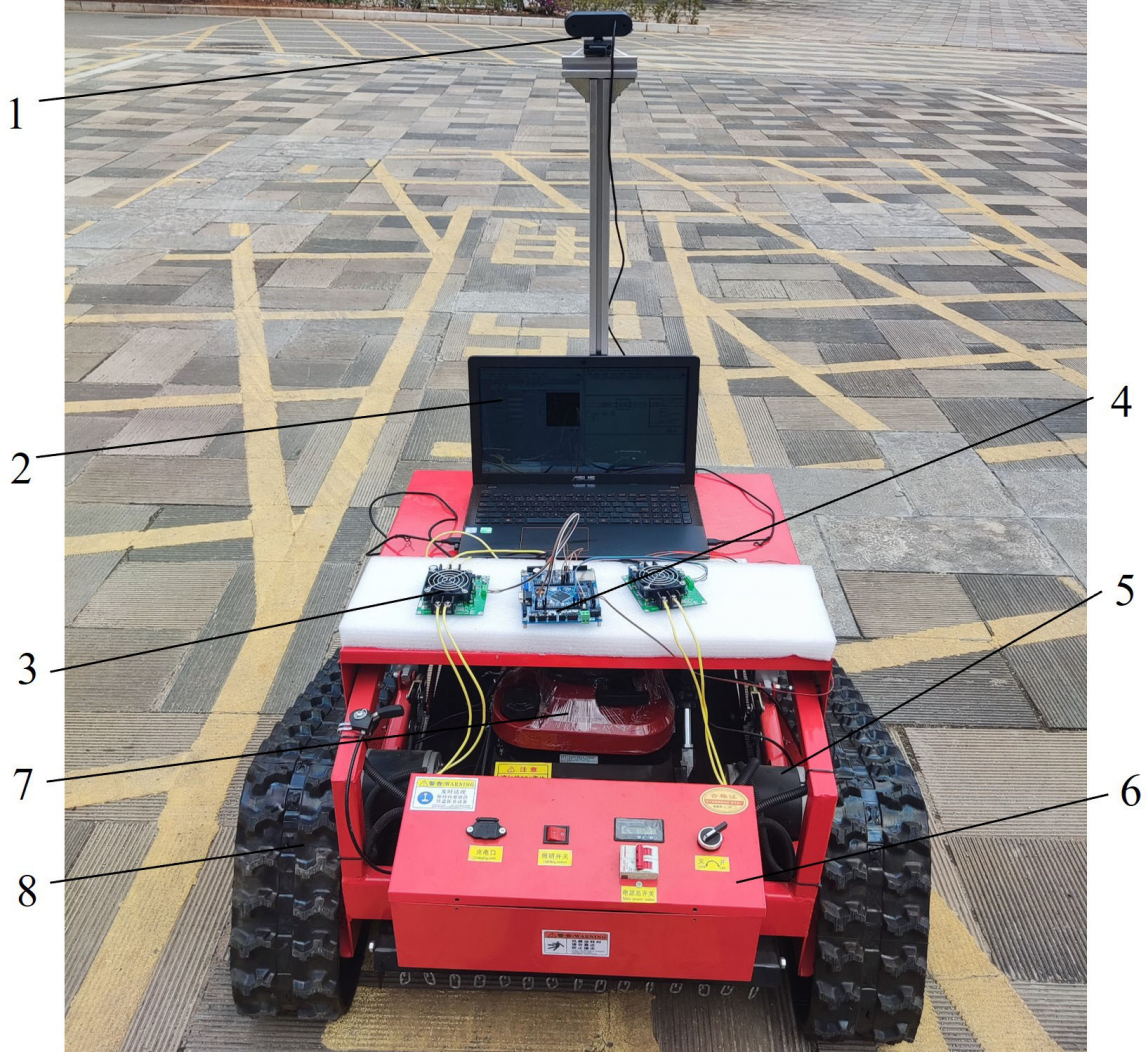
Robot experiment platform. 1. Camera; 2. laptop computer; 3. motor driver; 4. STM 32 controller; 5. motor; 6. control box; 7 gasoline engine; 8, track car.

Due to the complex environment in the Panax notoginseng shade house, its small plots, large slopes and high soil moisture, a triangular crawler chassis with an upland gap was used as the walking platform in this experiment. As shown in **Figure 9**, the crawler chassis has a running speed of 0–5 km/h and a maximum gradient of 60°. Considering the sowing and harvesting working speed, the walking speed was set to 1 m/s. **Table 3** reports the specific parameters of the crawler chassis used in the experimental platform.

**Table 3:** Specific parameters of crawler chassis.

Parameter	Performance
Dimensions (L × W × H)	930 mm × 900 mm × 600mm
Platform weight	140 kg
Running speed	0–5 km/h
Motor rated power	600W
Motor rated voltage	DC12 V
Motor speed	40–55 r/min
Rated current	50 A
Working method	Oil-electric hybrid
Track width	15cm
Track material	Rubber track with built in tension layer
Maximum grade	60°
Maximum load	150 kg
Generator power	1500 W
Engine	Gasoline engine
Equipment power	7.5 horsepower

**Figure 10:**
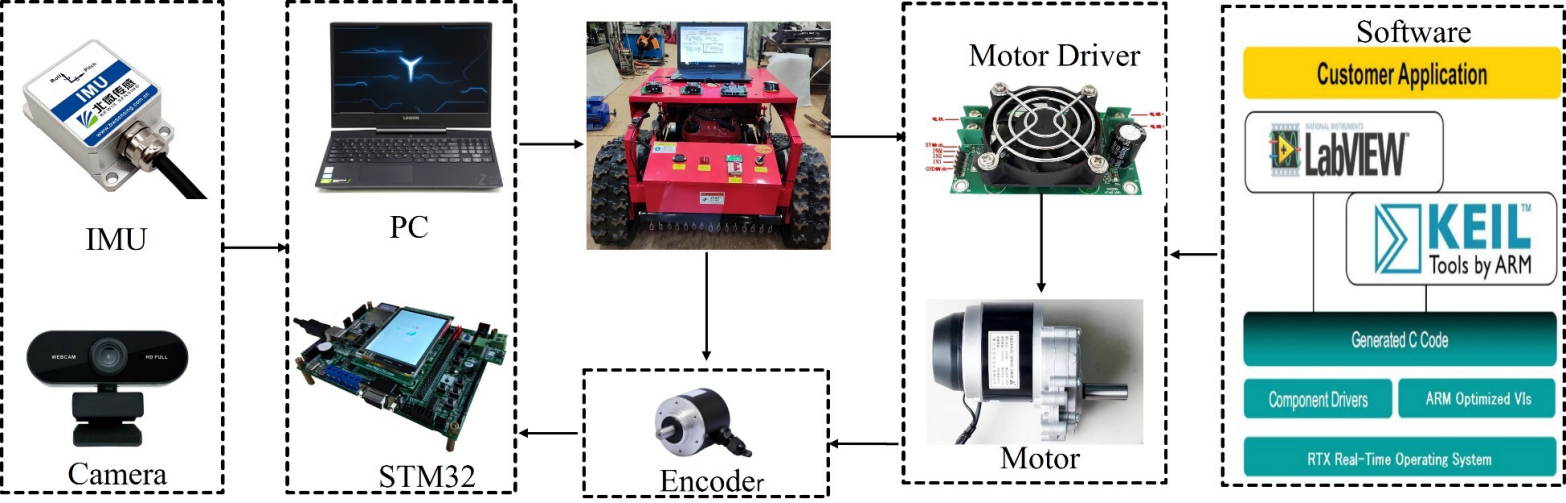
Experimental platform and control system.

We developed a LABVIEW program to control the robot platform using the ARM embedded software architecture of the STM32. This facilitated data monitoring. improved development efficiency, and reduced costs. **Figure 10** depicts the experimental platform and control system. The sensor module consisted of an encoder, an IMU, and a camera. A PC was used as the master computer to collect information from the camera and inertial navigation IMU sensors. The camera obtained information on the environment between the rows of the Panax notoginseng shade house, while the inertial navigation IMU collected data on the robot’s traverse, pitch, and yaw direction. The encoder measured the robot’s real-time velocity information and fed it to the robot’s underlying controller, the STM32F407. At the heart of the embedded system board was a high-performance 32-bit ARM Cortex-M4 processor with built-in high-speed memory and a rich set of I/O ports used to connect various external devices. The controller connected the motor driver, the encoder, and the host computer. The STM32 controlled the motor rotation through the motor driver based on the real-time speed information provided by the encoder, using a classical PID algorithm to achieve precise robot motion. The motor driver controlled the speed of the brushed DC motor using pulse width modulation (PWM). For the control process, the PC host computer processed the images captured by the camera in real-time and communicated with the STM32F407ZGT6 control module of the motion controller. The microcontroller sent PWM signals to the motor drive module, and the signals were amplified to drive the motor. At the same time, the inverter performed real-time AD sampling to provide over-current and over-voltage protection. The host computer communicated with the motion control module via the RS232 serial port, and the feedback information from the two motors was transmitted to the control module via the serial port to achieve closed-loop control.

### 3.2 Detection results of seven-fork

**Figure 11:**
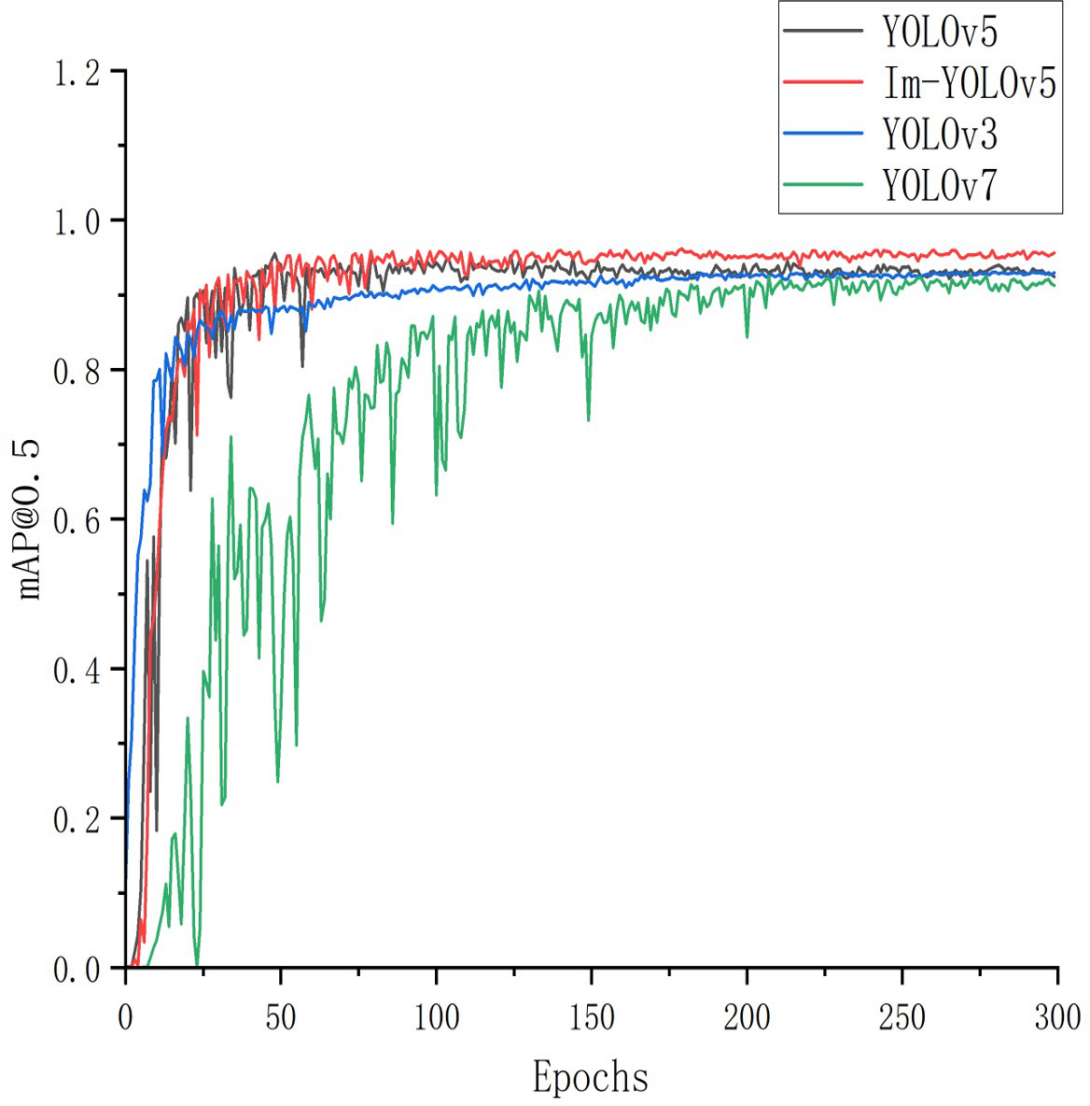
Accuracy variation of four object detectors.

We compared our improved target detection model with three of the most advanced, fastest detecting, and widely used models. mAP@0.5 was employed to plot the line graphs. **Figure 11** reveals that the YOLOv5 series model had an advantage in detecting the seven-forked roots. Although both YOLOv7 (Wang CY. et al., 2022) and YOLOv3 (Joseph and Ali, 2018) approached the YOLOv5 series models in terms of detection accuracy after 200 training rounds, their convergence rate was slow, and early training fluctuations were high. The Im-YOLOv5 model exhibited a significantly faster convergence rate and higher mAP@0.5 compared to the other three models.

#### 3.2.1 Comprision results with mainstream object detection models

To evaluate the performance of the detection models proposed in this paper, we trained the Im-YOLOv5, YOLOv3, YOLOv5s, and YOLOv7 algorithms under the same conditions and evaluated their performance on a test set. **Table 4** reports the performance comparison of the four detection models, revealing that the Im-YOLOv5 model exhibited the highest P, R, F, and mAP values and the lowest model weights and GFLOPs. Although the improved Im-YOLOv5 model had a slower FPS than YOLOv7 and YOLOv3, it demonstrated a better performance in identifying seven-forked roots with 94.9% detection accuracy considering all indicators. The Im-YOLOv5 model had the optimal detection performance, a with faster detection speed for a single image while meeting the real-time requirements. The results demonstrate that it can effectively meet the needs of inter-row robots for Panax notoginseng cultivation.

**Table 4:** Performance comparison of different object detection algorithms.

Model	P(%)	R(%)	F(%)	FPS	mAP (%)	Modelsize/MB	GFLOPs
Im-YOLOv5s	94.2	92.0	93.1	106.4	94.9	7.5	6.3
YOLOv5s	91.0	91.4	91.2	83.3	93.1	14.4	15.8
YOLOv3	90.2	91.7	90.9	144.9	92.5	17.4	13.0
YOLOv7	88.3	89.4	88.8	125.0	91.3	12.3	13.2

P, comparison of accuracy; R, recall; F, harmonic average; FPS, frame rate; m, mean average precision; GFLOPs, giga floating-point operations per second.

#### 3.2.2 Comparison of detection performance of improved model algorithm

The improved Im-YOLOv5 model reduces the number of parameters by replacing the MobileNetv3 network with the ECA attention mechanism module. This allows the model to focus more on the target for feature extraction and less on the roots of the seven-forks that are further away and beyond the sides. To evaluate the detection performance of the Im-YOLOv5s model in this study, we verified the trained model using 180 test set images and 3 test videos for detection accuracy and speed. The Im-YOLOv5s detection model achieved a 47.9% reduction in weight size, from 14.4 MB to 7.5 MB (**Table 5**). In addition, the frame rate and average accuracy increased by 27.7% and 1.9%, respectively. These improvements in detection performance reduced model inference time and increased model accuracy.

**Table 5:** Indexes before and after model improvement.

Model	P(%)	R(%)	FPS	mAP(%)	Model Size/MB
YOLOv5s	91.0	91.4	83.3	93.1	14.4
Im-YOLOv5s	94.2	92.0	106.4	94.9	7.5

**Figure 12:**
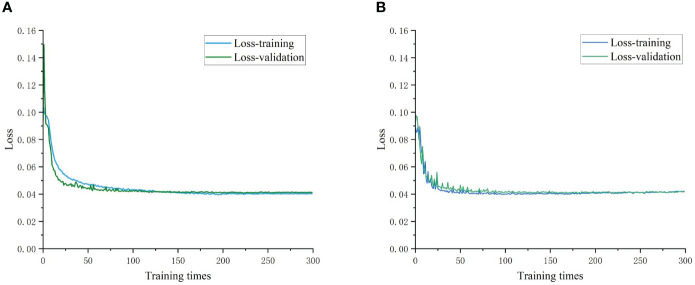
Loss iteration graph. **(A)** YOLOv5s loss iteration; **(B)** Im-YOLOv5s loss iteration.

**Figure 12** presents the model training and validation loss rate curves. We evaluated the recognition performance of both the YOLOv5s and Im-YOLOv5s models for the primary navigation features in terms of both model training and recognition results. The loss rate tended to stabilize as the number of iterations increased and eventually converged to a fixed value, indicating that the model achieved optimal results. The improved Im-YOLOv5s model demonstrated a better fit and generalization ability for the seven-fork root dataset while reducing the initial loss value.

#### 3.2.3 Experimental results in different scenarios

We utilized the Im-YOLOv5s model for target detection recognition in the test set and compared the experimental results of three scenes of the sowing, seedling, and harvested fields with high and low light intensity conditions. The results reveal a higher recognition accuracy for the sowing and seedling fields with a relatively clean background, reaching 95.7% and 95.2%, respectively (**Table 6**). However, the recognition accuracy of the cluttered unharvested Panax field was only 89.1%. Light intensity exerted a minor impact on the recognition results, with better recognition accuracy observed in both high and low light intensities.

**Table 6:** Experimental results under different backgrounds and light intensities.

Scenes	No. of photos	P(%)	R(%)	F(%)	mAP(%)
Land to be sown	48	92.6	93.7	93.1	95.7
Seedling land	58	92.8	92.4	92.6	95.2
Harvested land	77	85.1	88.1	86.6	89.1
High light intensities	62	92.1	92.5	92.3	93.4
Low light intensities	62	95.3	91.2	93.2	94.8

**Figure 13:**
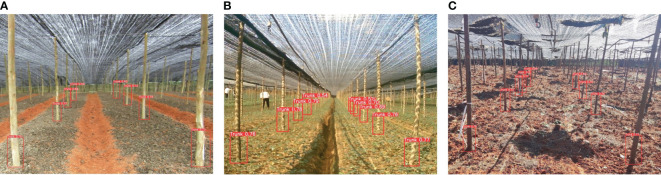
Recognition results in different scenes. **(A)** Land to be sown; **(B)** seedling land; **(C)** harvested land.

**Figure 14:**
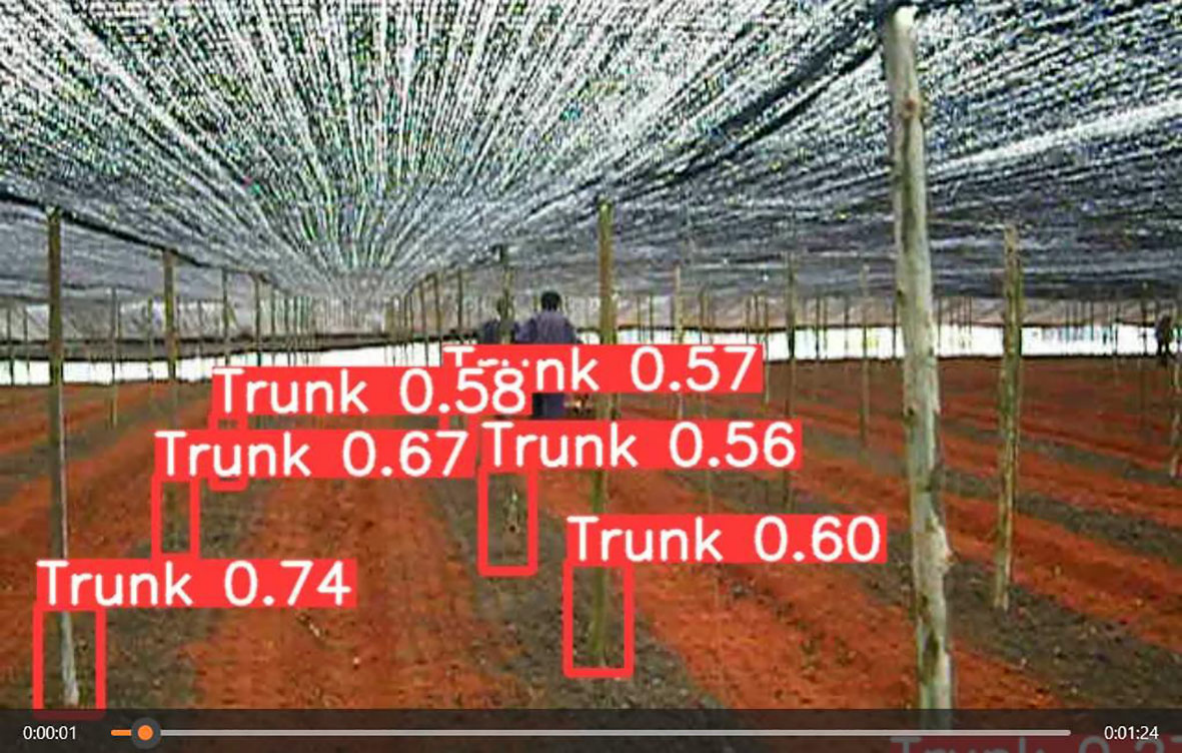
Video of actual environment.

**Figure 13** depicts the recognition results under different scenarios. Our proposed model can accurately identify the seven-fork roots under various scenarios and obtain the corresponding navigation feature information. **Figure 14** presents the results of the dynamic detection of the plot to be sown. Detecting moving targets is more challenging than the detection of stationary targets. In the video-based real-time detection, our model accurately detected the seven-forked roots between the two rows.

### 3.3 Navigation reference point acquisition experiment

**Figure 15:**
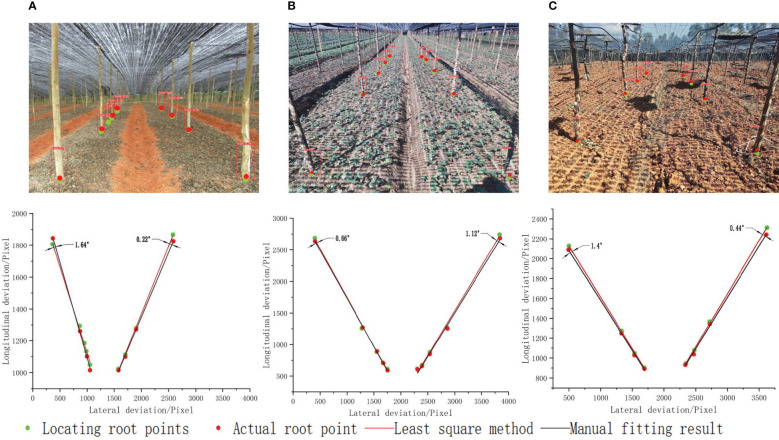
Seven branch positioning base points and actual root points in different scenes. **(A)** Land to be sown; **(B)** seedling land; **(C)** harvested land.

After identifying and framing the root of the seven-fork using deep learning, the coordinate values of the corner points of the rectangular bounding box were extracted. This included the coordinates of the upper left $P_{l}(x_{l},y_{l})$ and lower right $P_{r}(x_{r},y_{r})$ corner. The coordinates of the lower edge center were calculated $P_{i}(\frac{x_{r}-x_{l}}{2}+x_{l},y_{r})$ . We replaced the coordinates of the seven-fork root point with the center of the bottom edge, as shown in **Figure 15**, where the green and red points denote the seven-forks set as base points and the actual seven-fork root points marked by hand, respectively. To evaluate the accuracy of the reference point extraction, the manually marked reference points were selected as the evaluation criteria and separately fitted to a linear line. We defined the deviation of the line direction as the angle between the fitted line of the reference points extracted by the algorithm in this study and the fitted line of the manually marked points. The line extraction was considered correct if the absolute value of the deviation between the two was less than 4° (Zhai et al., 2022; Lai et al., 2023). After several experimental calibrations, the maximum and minimum deviations of the line direction for the three scenes were 1.64° and 0.22°, respectively. The results reveal that the proposed deep learning-based root point substitution method can accurately obtain the navigation reference lines.

### 3.4 Centerline extraction results

**Figure 16:**
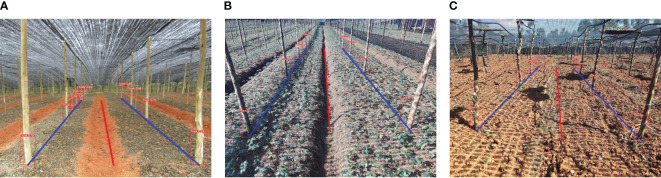
Results of navigation line extraction in different scenarios. **(A)** Land to be sown; **(B)** seedling land; **(C)** harvested land.

We experimentally verified the practical feasibility of the proposed inter-row navigation information acquisition method by selecting several pictures from the dataset collected by the external camera connected to the PC for testing. The images contained three scenes: sowing field, seedling field and harvesting field. The effect of the navigation line extraction for different scenes is shown in **Figure 16**, where the red line represents the navigation center line and the blue lines represent the left and right navigation reference lines.

## 4 Discussion

The success of target detection algorithms heavily depends on the extraction of navigation lines using deep learning methods. In this paper, we compared the proposed Im-YOLOv5s algorithm with existing detection methods for similar targets. **Table 7** reports the results. Aguiar et al. (2020) achieved an average accuracy of 52.98% and approximately 49 frames per second using SSD MobileNet-V2 on the USB accelerator for the detection of vineyard trunks using low-cost embedded devices. Ma et al. (2021) adopted a faster R-CNN target detection model to achieve 89.40% detection accuracy for kiwifruit tree trunk roots. Zhou et al. (2022) used a YOLO v3 model to detect orchard trunks with a detection accuracy of 92.11%. In this paper, we demonstrate that the proposed Im-YOLOv5s model strikes a balance between detection speed, model size, and accuracy. The improved model provides better detection performance, with a mAP value of 94.90%, approximately 106.4 frames per second, and a model size of 7.5 MB. Compared to current studies, the improved YOLOv5s model presents great progress in model size and detection time.

**Table 7:** Comparison of proposed method with existing methods.

Source	Method	Object	mAP(%)
Aguiar, A.S.P. [14]	SSD	Vineyard	52.98
Ma et al. [15]	Faster R-CNN	Kiwi trunk	89.40
Zhou et al. [17]	YOLO v3	Orchard trunk	92.11
Proposed method	Im-YOLOv5s	Seven-fork	94.90

Although the proposed method in this study can accurately extract the centerline of seven-fork rows in Panax notoginseng shade house, we came across several limitations. While the improved deep learning model enhances detection accuracy and speed, the field of view range and camera jitter can affect the detection accuracy rate of the seven-fork roots, which, in turn, affects the fitting error of the navigation line. For the complex and variable inter-row environment, relying solely on visual sensing to obtain navigation feature information may pose significant risks to the actual safety of robot operation. In future work, we plan to transfer the deep learning model and seven-fork row centerline extraction algorithm to a mobile robot platform and combine them with multi-sensor fusion technology to achieve automatic navigation in the semi-structured environment of Panax notoginseng shade trellis.

## 5 Conclusions

In this paper, we proposed a navigation line extraction method based on Im-YOLOv5s. By replacing the original backbone with a lightweight network architecture, MobileNetv3, and introducing the ECANet attention mechanism module, we improved the model’s recognition accuracy and robustness while reducing its weight size by 47.9%, increasing the frame rate by 27.7%, and improving the average accuracy by 1.9%. The algorithm efficiently and accurately extracted information on the seven-fork roots in the shade trellis with an average detection accuracy of 94.9%, and was resilient to light and shadow disturbances. We located the coordinates of the root point according to the bottom edge midpoint of the outer rectangular box of the detected seven-fork roots and used the least squares method to fit the navigation reference line on both sides. The maximum deviation of the row direction was 1.64°, which met the criteria for navigation line extraction. We then used the detected bilateral column line as the navigation reference line and extracted the middle navigation line using the angle tangent formula to determine the robot’s forward direction. The proposed method provides a technical reference for inter-row path planning.

## Data availability statement

The original contributions presented in the study are included in the article/supplementary material. Further inquiries can be directed to the corresponding author.

## Author contributions

YT designed the lightweight model and trained the model, YT and PL collected the images of Panax notoginseng shade house, JJ guided the experiment, YT designed hardware and software for the robotics platform and wrote the original draft, CLW, WS, QHL, JJ and YW reviewed and edited the paper. The authors declare that the research was conducted in the absence of any commercial or financial relationships that could be construed as a potential conflict of interest. All authors contributed to the article and approved the submitted version.
